# Origin, Fate, and Function of Epicardium-Derived Cells (EPDCs) in Normal and Abnormal Cardiac Development

**DOI:** 10.1100/tsw.2007.294

**Published:** 2007-11-12

**Authors:** Heleen Lie-Venema, Nynke M. S. van den Akker, Noortje A. M. Bax, Elizabeth M. Winter, Saskia Maas, Tuija Kekarainen, Rob C. Hoeben, Marco C. deRuiter, Robert E. Poelmann, Adriana C. Gittenberger-de Groot

**Affiliations:** ^1^ Department of Anatomy and Embryology, Leiden University Medical Center, Leiden, The Netherlands; ^2^ Virus and Stem Cell Biology Lab., Department of Molecular Cell Biology, Leiden University Medical Center, Leiden, The Netherlands

**Keywords:** embryo, proepicardial organ, epicardium, epicardium-derived cell (EPDC), migration, Ets transcription factors, cardiac looping, coronary artery, cardiac valve, myocardium, Purkinje fiber, congenital heart defects

## Abstract

During heart development, cells of the primary and secondary heart field give rise to the myocardial component of the heart. The neural crest and epicardium provide the heart with a considerable amount of nonmyocardial cells that are indispensable for correct heart development. During the past 2 decades, the importance of epicardium-derived cells (EPDCs) in heart formation became increasingly clear. The epicardium is embryologically formed by the outgrowth of proepicardial cells over the naked heart tube. Following epithelial-mesenchymal transformation, EPDCs form the subepicardial mesenchyme and subsequently migrate into the myocardium, and differentiate into smooth muscle cells and fibroblasts. They contribute to the media of the coronary arteries, to the atrioventricular valves, and the fibrous heart skeleton. Furthermore, they are important for the myocardial architecture of the ventricular walls and for the induction of Purkinje fiber formation.

Whereas the exact signaling cascades in EPDC migration and function still need to be elucidated, recent research has revealed several factors that are involved in EPDC migration and specialization, and in the cross-talk between EPDCs and other cells during heart development. Among these factors are the Ets transcription factors Ets-1 and Ets-2. New data obtained with lentiviral antisense constructs targeting Ets-1 and Ets-2 specifically in the epicardium indicate that both factors are independently involved in the migratory behavior of EPDCs. Ets-2 seems to be especially important for the migration of EPDCs into the myocardial wall, and to subendocardial positions in the atrioventricular cushions and the trabeculae.

With respect to the clinical importance of correct EPDC development, the relation with coronary arteriogenesis has been noted well before. In this review, we also propose a role for EPDCs in cardiac looping, and emphasize their contribution to the development of the valves and myocardial architecture. Lastly, we focus on the congenital heart anomalies that might be caused primarily by an epicardial developmental defect.

### INTRODUCTION

Early in vertebrate development, heart morphogenesis is initiated by the fusion of two cardiogenic primordia of the splanchnopleuric mesoderm[1,2]. After fusion at midline, the early embryonic heart consists of a myocardial tube lined with endocardium, with the cardiac jelly containing extracellular matrix molecules, in between. After the first looping stages, the cardiac jelly becomes unevenly distributed and is invaded by mesenchymal cells derived from the endocardium by epithelial-mesenchymal transformation (EMT). Thus, endocardial cushions are formed at the future atrioventricular junction and in the outflow tract. At this point in development, two extracardiac sources start to deliver cells in and onto the heart tube. Migrating cells originating from the cardiac neural crest invade the heart via the aortic arches[3,4]. They contribute to the formation of the aorticopulmonary septum and outflow tract endocardial cushions[5,6,7]. Cardiac neural crest cells (CNCs) also enter the inflow portion of the heart via remnants of the dorsal mesocardium. Here they migrate into the dorsal mesenchymal protrusion forming the vestibular spine, from where they contribute to the base of the atrial septum and the condensed mesenchyme that is forming the membranous part of the ventricular septum[8,9,10]. It has been suggested that CNCs contribute to the heart mostly as instructive cells, rather than as constructive cells, because the majority of the CNCs are destined for apoptosis[4]. For a detailed review on the role of CNCs in cardiac development, we refer to the contribution of Snider et al.[11] in this issue of *TheScientificWorldJOURNAL*.

The second population of cells that have been described as extracardiac contributors are derived from the epicardium. These so-called epicardium-derived cells (EPDCs) have both constructive and instructive roles in heart morphogenesis, which will both be discussed in this review.

### DO EPDCs ORIGINATE FROM AN EXTRACARDIAC SOURCE?

Whether EPDCs may be regarded as true extracardiac contributors, as the neural crest cells undoubtedly are, is a matter of debate in view of recent new concepts on the spatiotemporal addition of cells from the heart-forming fields. The primary heart tube derives from the first heart-forming field[12]. A secondary heart field subsequently adds cells to both the outflow and inflow tract. Confusingly, this second heart field is divided into an outflow tract contribution referred to as the secondary or anterior heart field[13,14], while at the inflow tract, the posterior heart field can be discerned[15]. This posterior heart field is located at the site where the sinus venosus enters the pericardial cavity and gives rise to the cardiomyocytes that will form the central part of the conduction system and to the proepicardial organ (PEO)[15]. The description of the posterior heart field as the site from which the PEO originates fits well with other descriptions of PEO development[16]. The discrimination between the primary, anterior, and posterior heart-forming fields is based on both spatial and temporal characteristics. However, it may well be conceived that these heart fields develop as a spatiotemporal continuum, which is depicted in Fig. 1. As the PEO appears to originate from a part of this continuous region of heart-forming fields, the idea of an extracardiac origin of the epicardium needs to be reconsidered.

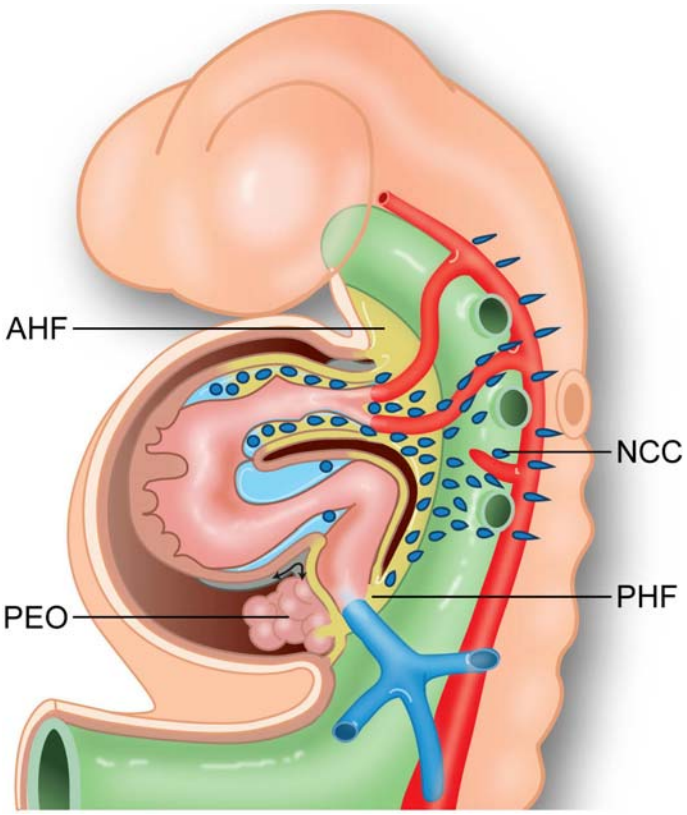

**FIGURE 1.** Schematic picture of the continuum of the primary (brown and blue) and secondary (yellow) heart-forming fields. The secondary heart-forming field can be divided into the anterior heart field (AHF), at the outflow region of the heart, and the posterior heart field (PHF), at the inlet region of the heart. The PEO, the cauliflower-like protrusion of the sinus venosus into the pericardial cavity, develops as a part of the posterior heart field and can thus be regarded as originating from a cardiac source. The neural crest cells (NCC) are true extracardiac contributors to heart development as they migrate from the neural crest along the arterial and venous pole into the heart, to the blue regions originating from the primary heart field. Adapted with permission from Poelmann and Gittenberger-de Groot[168].

### PROEPICARDIAL DEVELOPMENT

Early during development, the epicardium forms from the splanchnopleural mesoderm of the posterior heart field and is initiated by the formation of a villous structure, called the proepicardial serosa, proepicardium, or proepicardial organ (PEO). The PEO protrudes from the pericardial mesothelium covering the sinus venosus into the pericardial cavity in the direction of the tubular, looped heart[1,17,18,19,20,21]. Because proepicardial villi harbor epithelial, mesenchymal, and endothelial (precursor) cells[22,23,24,25], we prefer to call it the PEO. Although the first descriptive studies of the PEO were performed in chicken and quail, PEO formation is common to a range of vertebrate species, including axolotl[26], dogfish[27], mouse[28], rat[29], and treeshrew[30]. Both in mammalian and avian embryos, the PEO is formed initially pairwise as bilateral and symmetrical structures on the transverse septum and sinus venosus, respectively. In chicken, the left PEO anlage does not persist, whereas the right PEO will develop into the cauliflower-like protrusion[31,32].

Whereas little is known about the factors that induce initial PEO formation, the differentiation of the PEO into a migrating mesothelium distinct from the cardiac (precursor) cells of the sinus venosus wall is known to be regulated by bone morphogenetic protein (BMP) and fibroblast growth factor (FGF) signaling[16,33].

### EPICARDIAL DEVELOPMENT

Migration of proepicardial cells to the naked heart tube, where they initially form a mesothelial outside covering of the myocardium, was long believed to show distinct species differences. In amphibian and avian embryos, the proepicardial cells predominantly migrate along extracellular matrix tissue bridges[20,21,34]. In mammalian embryos, but also in fish, proepicardial cell clusters were observed to detach from the proepicardial villi and float freely through the pericardial cavity until they attach to the myocardium[27,28,30,31]. Amphibians seem to employ both ways for transfer of proepicardial cells across the pericardial cavity to the myocardium[26]. Proof that the species differences are not so strict as thought initially comes from the recent description that, in the rat, direct attachment through tissue bridges is similar to that in birds[35]. Furthermore, in quail embryos, isolated epicardial patches on the myocardium indicative of previously existent free-floating vesicles were also observed[21].

After attachment to the myocardial surface, the cells start to migrate laterally, until the complete heart is enveloped by the epicardium. In chicken and quail embryos, the proepicardial cells transverse the pericardial cavity and contact the myocardium at the dorsal side of the atrioventricular canal at Hamburger and Hamilton[36] stage 17[21,22,25]. The epicardial cells then spread radially, and start to circumvent the atrioventricular region, the inner curvature, and dorsal side of the outflow tract, until eventually the last uncovered areas are the left atrium and parts of the distal outflow tract. The heart is completely enveloped at HH26 (ED5)[21]. The proepicardial vesicles in mammalian embryos display a similar spatiotemporal pattern in PEO attachment and outgrowth of the epicardial sheet[29], which might well be explained by the existence of bird-like extracellular matrix bridges[35]. Regional differences in the molecular make-up of the extracellular matrix attachment sites along the heart tube are likely to play a role as well. Transplantation experiments in which quail PEOs were transplanted to the pericardial cavity, adjacent to the naked heart tube, showed that attachment of the quail PEO never succeeded when the donor PEO was placed next to the developing ventricular region[25]. Factors important for migration and adhesion of the proepicardium and epicardium to the underlying myocardium described to date are vascular cell adhesion molecule 1(VCAM-1), $\alpha 4$ integrin, and Tbx5[37,38,39,40,41].

The epicardium surrounding the arterial pole has been described not to originate from the PEO, but from the coelomic/pericardial mesothelium at the site where the aortic sac leaves the pericardial cavity[42,43,44]. Normally, this cell population will form the outer mesenchymal layer of the arterial pole within the pericardial cavity (the arterial epicardium), whereas the proepicardial epicardium will cover the myocardial outflow tract[42] (the cardiac epicardium). In PEO-ablated quail embryos, the arterial epicardial cells may migrate further “upstream” and also cover the myocardium of the most distal outflow tract[43]. The arterial epicardium differs from the cardiac epicardium in its lower expression of retinaldehyde-dehydrogenase2 (RALDH2) and vimentin, and its more limited potential for EMT[44]. Whether there is a functional difference between the cardiac epicardium and the arterial epicardium is as yet unclear.

Lateral migration of the cardiac epicardial sheet is probably driven by both migratory and proliferative processes. Once the heart is covered by the epicardial mesothelial sheet, EPDCs start to form by EMT[45,46]. Mesenchymal EPDCs thus delivered to the subepicardial extracellular matrix will form the subepicardium. The thickness of the subepicardium will eventually vary according to the underlying heart structure that is covered, and may vary between species. In chicken embryos, it is relatively thin (one to three cells) at the atrial and ventricular myocardium, whereas it is thick in the atrioventricular sulcus where abundant EMT will provide for all the EPDCs needed for coronary formation[45].

Factors that have been implied in the regulation of EMT of the epicardial mesothelium are the homeobox transcription factors Slug and Snail[47,48,49,50], the transcription factor Wilms tumor suppressor protein WT-1[51,52,53], the Ets transcription factors Ets-1 and Ets-2[54], the adhesion molecules E-cadherin[49], α4 integrin[40], and paracrine growth factors, such as FGF[55], transforming growth factor TGFβ[55,56,57,58], platelet-derived growth factor (PDGF)[59,60,61], and vascular-endothelial growth factor (VEGF)[55].

### MIGRATORY FATE OF EPDCs

From the subepicardium, mesenchymal EPDCs will form migratory processes (see Fig. 2g) and start to invade the myocardium in a spatiotemporally regulated fashion, in which factors expressed by the underlying myocardium define the permissiveness for EPDCs[25]. In chicken embryos, EPDCs will invade the myocardium starting from the inner curvature at HH19 (ED3), subsequently followed by the atrioventricular canal, atria, and ventricles at stage 23/24, and finally the outflow tract from HH30 (ED6.5) onwards[25]. EPDCs migrate into the underlying myocardium in a tangential pattern[62], but do not seem to spread over large distances; most EPDCs are retained within that part of the heart that is directly underneath the local area of subepicardium from which they originated[25].

Once within the myocardium, EPDCs will move to their final destinations. Although the precise temporal regulation of EPDC migration has not been defined, two influxes of EPDCs into the chicken embryonic heart have been identified: the first between HH19–31 (ED3–7), directly following the process of EMT and formation of the subepicardium, and the second between HH31–43 (ED7–17)[62]. EPDCs from the first influx will readily take up subendocardial positions, both in the atrium and in the ventricles via small fenestrations in the myocardial layer that enable easy contact between the epicardium and endocardium, and will migrate into the myocardial interstitial spaces of atrium and ventricles. EPDCs from the second wave will migrate into the atrioventricular cushions. From epicardial quail-chicken chimeras, we know that at embryonic stage HH35 (ED9), most EPDCs have taken up their final positions: around the coronary arteries as smooth muscle cells (SMCs) and fibroblasts[23,42,45,63], in the atrioventricular cushions[42,62], in the subendocardium of the ventricular trabeculae and atria[62], and in the ventricular myocardium as interstitial fibroblasts[25,42,62].

Factors regulating migration of the EPDCs to their final destiny are PDGF-B and PDGFRβ[61], Tbx5[41], thymosin β4[64], and Ets transcription factors (this review, see below).

### THE ROLE OF ETS TRANSCRIPTION FACTORS IN EPDC MIGRATORY BEHAVIOR

Ets transcription factors are important for epicardial development as was revealed by the morphology of chicken embryos in which both Ets-1 and Ets-2 were down-regulated simultaneously by retroviral antisense Ets treatment[54]. Transduced embryos suffered from coronary maldevelopment and a thin ventricular myocardium, which could be related to defective EMT of the epicardium. The genes that are known to have and/or use Ets binding sites encode transcription factors, cell-cycle regulators, matrix-degrading proteinases, cytokeratins, cell adhesion molecules, and growth factors[65]. This experimental section focuses on how Ets-1 and Ets-2 are involved in the capacity of EPDCs to reach their destinies in the developing heart. For technical data, we refer to the Materials and Methods Supplement to this review.

To unravel which of the two Ets transcription factors was responsible for the cardiac defects in the antisense Ets-treated embryos, we down-regulated Ets-1 and Ets-2 expression separately or concomitantly by lentiviral constructs carrying an antisense Ets sequence in conjunction with the GFP reporter gene. The antisense constructs were specific for either Ets-1 (asEts-1), Ets-2 (asEts-2), or both (asEts-1/2). We analyzed EPDC behavior after transduction with the antisense Ets lentiviruses and the GFP controls, both *in vitro*, in proepicardial quail explants, and *in vivo* in proepicardial quail-chicken chimeras.

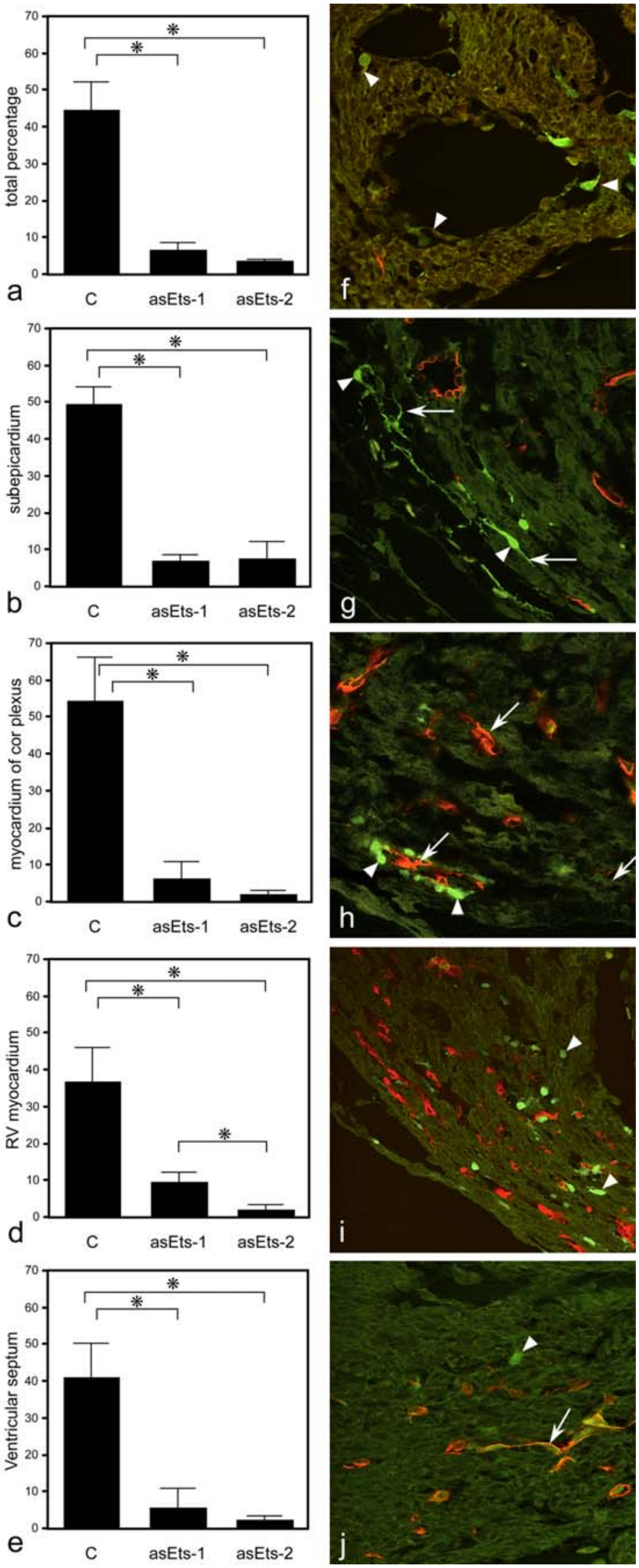

**FIGURE 2.** Ets-1 and Ets-2 transcription factors are important for the migratory potential of EPDCs. Quail-chicken chimeras (HH35; ED 8.5) in which the transplanted PEO was transduced with a lentiviral construct harboring either a CMV-GFP, a CMV-GFP-asEts-1, or a CMV-GFP-asEts-2 expression cassette were analyzed by light and confocal microscopy. Compared to the EPDCs that were transduced with the control CMV-GFP lentivirus, EPDCs expressing either GFP-asEts-1 or GFP-asEts-2 were found in severely reduced total numbers in the heart. (a) Similar statistically significant (*) differences were found between EPDCs harboring the control and antisense constructs in selected regions of the heart: the subepicardium (b,g), the myocardium in which the coronary plexus develops (c,h), the myocardial wall of the right ventricle (d,i), and the myocardium of the ventricular septum (e,j). The Y-axis in the left panels represents the number of GFP-positive cells as a percentage of the total amount of quail-derived QCPN-positive cells. That GFP expression did not affect the normal migration potential of the EPDCs is shown in green in the confocal photomicrographs in the right panels (f–j). GFP-positive EPDCs were observed at subendothelial trabecular sites (arrowheads in f), in the subepicardium (arrowheads in g) where the GFP marker highlights the migratory processes of the mesenchymal EPDCs (arrows in g), in the walls of the coronary arteries (arrowheads in h), as interstitial cells of the ventricular myocardial wall (arrowheads in i), and in the ventricular septum (arrowheads in j). Although the transduced proepicardial explants contained a tiny piece of liver for the delivery of endothelial (precursor) cells, GFP expression hardly ever colocalized with the quail-specific endothelial marker QH1 (red; arrows in h). Only in the ventricular septum occasional double-staining of GFP-positive endothelial cells was observed (yellow signal indicated by an arrow in j).

Hardly any GFP-positive cells were observed in proepicardial cultures transduced with the asEts-1/2 lentivirus (0.5 ± 0.6% GFP-positive cells, n = 6). Apparently, cells in which both Ets-1 and Ets-2 were down-regulated did not manage to survive and/or proliferate, and were overgrown by the untransduced GFP-negative cells. Down-regulation of either Ets-1 or Ets-2 did not have such a dramatic effect. In the cultures transduced with the asEts-1 and asEts-2 lentivirus, transduction percentages were observed that were not significantly different to those obtained for the control lentivirus (control GFP, 78 ± 8%, n = 8; asEts-1, 57 ± 14%, n = 8; asEts-2, 57 ± 11%, n = 7). The PEO explant cultures contained only few endothelial (precursor) cells, and none of these cells was GFP positive. Both migratory behavior and differentiation into SMCs were the same in cells transduced with either the GFP-asEts-1 or GFP-asEts-2 virus, or the GFP-control. Apparently, lateral migration and *in vitro* differentiation of the PEO cells was not disturbed by Ets-1 or Ets-2 down-regulation. Cell morphology and radial outgrowth of the PEO cells also did not differ between GFP-transduced and -untransduced controls.

In contrast to the outcome of the *in vitro* experiments, the migratory behavior of proepicardial cells was severely affected after asEts-1 or asEts-2 treatment *in vivo* in quail-chicken chimeras. The proepicardial quail-chicken chimera model was used because in this model, the presence and nearly undisturbed outgrowth of the endogenous PEO ensures normal heart development and gives the opportunity to study (manipulated) quail PEO-cell behavior in a relatively normal physiological setting.

asEts-1/2–treated PEOs failed to graft in all cases (n = 20) whereas, normally, approximately 50% of the PEO transplantations gives rise to bona fide proepicardial quail-chicken chimeras in our hands. Chimeras containing PEO grafts treated with either asEts-1 or asEts-2, or the CMV-GFP control virus, were obtained in almost normal proportions (in total 43 cases out of 83 chimerization experiments). The grafted EPDCs were visualized in stage HH35 (ED9) chimeras and ratios of GFP- vs. QCPN-positive cells were determined in diverse segments of the heart (see Fig. 2 and text below). The transduction efficiency of the control CMV-GFP lentivirus reached similar levels in the proepicardial outgrowth derivatives *in vivo* as *in vitro*; in the control GFP chimeras, 45% of the quail-derived QCPN-positive cells was GFP positive. Only few quail-derived QH-1–positive endothelial cells were transduced with the control virus; most of the GFP-positive cells were true EPDCs.

The total proportion of GFP-positive cells in the asEts-treated proepicardial chimeras was significantly lower than in chimeric GFP controls; more than 6-fold in asEts-1 chimeras and more than 12-fold in asEts-2 chimeras (Fig. 2a). There were four specific cardiac areas suitable for quantitative analysis: the subepicardium (Fig. 2b,g); the myocardium containing the coronary plexus (Fig. 2c,h); the myocardium of base, moderator band, and lateral wall of the right ventricle (Fig. 2d,i); and the interventricular septum (Fig. 2e,i). In all these regions, the ratio between GFP- and QCPN-positive cells was significantly lower in the asEts-treated embryos than in the GFP controls (Fig. 2b–e). In general, there were lower levels of GFP-positive cells in the myocardium of asEts-2 chimeras than in that of asEts-1 chimeras, but only in the myocardium of the right ventricle was there a significant difference between the migration potential of asEts-1– and asEts-2–treated EPDCs (Fig. 2d). Notably, in the asEts-2 chimeras, the highest proportion of GFP-positive cells was observed in the subepicardium. This may indicate that asEts-2–treated cells are retained in the subepicardium because they have more difficulty migrating into the underlying myocardium than asEts-1–treated EPDCs.

In addition to the statistically significant differences described above, we found that asEts-2–treated EPDCs never reached subendocardial positions in the atrioventricular cushions in the two embryos where GFP-negative, quail-derived, QCPN-positive cells were present at these locations. The presence of lentivirally expressed asEts-1 did not obstruct EPDC migration to the subendocardial cushion in three embryos, although only 4% of the QCPN-positive cells also expressed GFP. In two control embryos, GFP-positive EPDCs (20% of the quail-derived cells) could be found in the right atrioventricular cushion. Similarly, in the control chimeras, GFP-positive EPDCs were present in the trabeculae of the left and right ventricles (RV in three embryos; GFP vs. QCPN ratio 59 ± 17% SEM; LV in two embryos GFP vs. QCPN ratio 50%), as is shown in Fig. 2f. Grafted EPDCs were found in the trabeculae of single asEts-1 and asEts-2 specimens, but these were never GFP positive.

In conclusion, both Ets-1 and Ets-2 have a role in the migratory capacity of EPDCs *in vivo*. The presence of either Ets-1 or Ets-2 is sufficient for adequate attachment of the PEO to the myocardial surface, but when both transcription factors are absent (as in the PEOs transduced with asEts-1/2), this function cannot be taken over by other members of the Ets transcription factor family. Although lateral migration does not seem to be affected by antisense Ets treatment, the migration into the myocardium is hampered when either Ets-1 or Ets-2 is down-regulated. Of these two Ets transcription factors, the more severe disturbance in EPDC migration after Ets-2 down-regulation indicates that Ets-2 may predominantly regulate EPDC fate, especially in the ventricular wall and at subendocardial positions. To get more insight into the *in vivo* effect of (epicardial) Ets down-regulation on cardiac development, it would be worthwhile to study the cardiac morphology of the Ets-1 and Ets-2 knockout mice[66,67,68] in detail, and to generate quail-chicken chimeras in which lentivirally transduced antisense Ets-1 or Ets-2 PEOs replace the endogenous ones.

### FUNCTION OF EPDCs IN CARDIAC DEVELOPMENT

Proof for the functional significance of EPDCs in cardiac development comes from several experimental models in which epicardial development has been disturbed either mechanically[43,69,70,71,72] or genetically[37,38,54,73,74,75,76,77]. Thus, EPDCs have been inferred to play a crucial role in the development of the coronary vasculature, the atrioventricular valves, myocardial architecture, and the peripheral conduction system, i.e., the Purkinje fibers. These distinct roles of EPDCs in cardiac development are discussed below and in Fig. 3 a schematic overview is depicted. In this figure and in the text, we only mention the factors and signaling routes that are known to be involved in these processes; for a more detailed discussion of these factors, we would like to refer to several recent reviews on the topic[78,79,80,81].

#### Looping

Cardiac looping is a process consisting of sequentially (1) rightward bending of the initially straight heart tube (dextral looping), (2) transformation of the thus-formed C-shaped loop into an S-shaped loop, and (3) the later morphogenic events leading to position changes of the outflow tract with respect to the atria, in chicken embryos from stage HH9–24 (ED1.5–4)[82]. Because dextral looping is the first morphological event showing left-right asymmetry during embryonic development, many studies have concentrated on the mechanics and genetic factors involved in this initial looping phase (for review see, e.g., [83,84,85]). Much less is known about the subsequent looping phases and, until now, practically no attention has been paid to a possible role of the epicardium or EPDCs in cardiac looping. However, the inner curvature, which is obviously involved in the cellular movements during cardiac looping, is the first site of EPDC and endothelial (precursor) cell entry into the myocardium[25] at a developmental stage just preceding the last phase of looping (HH20–24; ED3–4). We also have several indications that disorders related to the last looping phase may arise with defects in epicardial development. As is shown in Fig. 3b, embryos with ablation of the PEO show a far-too-wide inner curvature[43]. In embryos with disturbed epicardial EMT due to Ets transcription factor down-regulation, we observed several specimens with a double-outlet right ventricle, which is an indication of disturbances in the remodeling of the inner curvature during the looping stage[86,87,88]. In several mouse models, a wider inner curvature or defects related to abnormalities in the third looping phase have been shown to coincide with absence of the epicardium. Examples are the knockout mice with mutant or deficient genes for $\alpha$4 integrin[38], retinoid X receptor RXR$\alpha$[89], zinc finger transcription factor GATA4[77], the GATA-co factor FOG-2[74], and TGF$\beta$2[90].

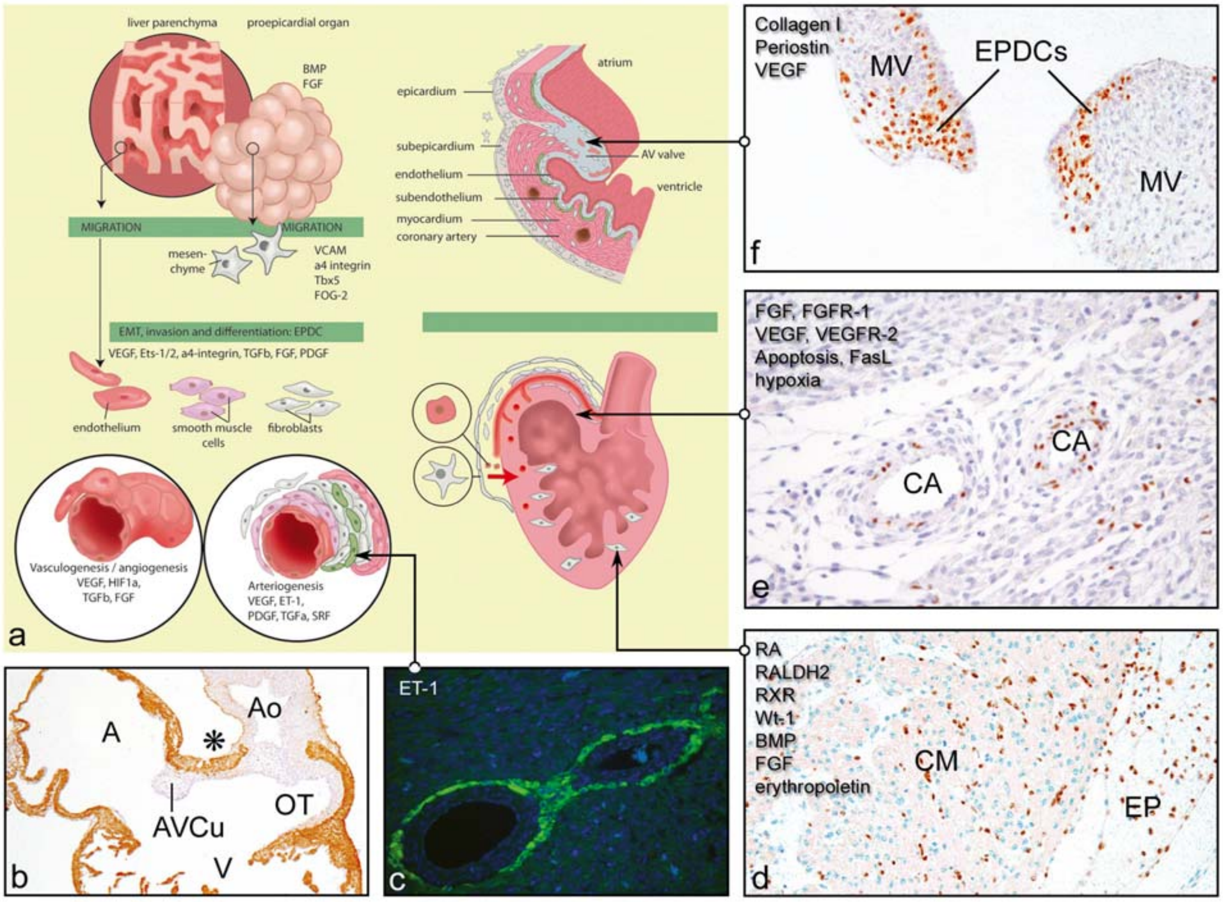

**FIGURE 3.** Schematic figure of EPDC fate and function. (a) Together with the development of the PEO, endothelial precursor cells of the liver region travel along with the proepicardial cells to the naked heart tube. After migration and epithelial-mesenchymal transformation of the epicardium, the subepicardial mesenchyme is formed, and EPDCs start to migrate into the underlying myocardium (a,f) where they differentiate into SMCs and fibroblasts. Counterclockwise, the photomicrographs (b–f) indicate their migratory fates and functions. Factors known to be involved in these developmental processes are listed in the left upper corner of the photomicrographs (for references, see text). (b) Migration into the myocardium starts in the inner curvature, where EPDCs may well have a function in the late looping process. This photomicrograph shows a far-too-wide inner curvature after mechanical ablation of the PEO ([43], used with permission from the authors). (c) EPDCs induce the formation of the peripheral conduction system; Purkinje fibers, shown here after immunofluorescent staining with the EAP-300 antibody, will not develop properly in epicardially inhibited embryos ([71], used with permission from the authors). (d) In a proepicardial quail-chicken chimera, tracing with the quail-specific nuclear marker QCPN reveals that a large proportion of the cells of the ventricular myocardial walls are EPDCs ([69], used with permission from the authors). Here, they contribute to myocardial architecture by expression of procollagen I and the extracellular matrix protein periostin (for references see text). (e) This photomicrograph of a quail-chicken chimera demonstrates that in the coronary arteries, EPDCs constitute the SMCs and fibroblasts that stabilize the vessel walls. (f) In the developing atrioventricular valves, many of the fibroblasts in the valve leaflets are epicardium derived, as is shown in this section of a proepicardial quail-chicken chimera ([69], used with permission from the authors). A, atrium; Ao, aorta; AVCu, atrioventricular cushion; CA, coronary artery; CM, myocardium; EP, (sub)epicardium; MV, mitral valve; V, ventricle.

EPDCs in the inner curvature that are involved in cardiac looping will be subjected to signals induced by hemodynamic forces and shear stress via the cytoskeleton. For a review on this topic, we would like to refer to the contribution of Linask and VanAucker[85] to this issue of *TheScientificWorldJOURNAL*.

### Coronary Development

In contrast to the limited numbers of studies indicating a role for EPDCs in looping, a vast amount of evidence for the crucial role of EPDCs in coronary development is available. Development of the coronary vasculature has been studied most extensively in chicken and quail embryos, and closely resembles mammalian coronary development[91]. In avian species, coronary vessels start to form from developmental stage HH23–25 (ED3.5–4.5) onward. Liver-derived endothelial precursor cells traveling along with the PEO and the epicardium enter the subepicardial mesenchyme and, by vasculogenesis, give rise to the early endothelial capillaries of the coronary plexus[22,23,24,25,92]. Protein factors implied in the regulation of early endothelial cell migration, proliferation, primary tube formation, and subsequent angiogenesis are listed in Fig. 3a and have been reviewed[93,94]. The initial endothelial vessel network is in open contact with the sinus venosus and starts growing from the dorsal side of the subepicardial mesenchyme of the atrioventricular sulcus[24,95]. From here, it encircles the atrioventricular groove towards the ventral side and is connected to the sinus venosus at this time point. Although smooth muscle components have been observed at stage HH27 (ED5)[24], thorough stabilization of the vascular network by SMCs and fibroblasts takes place after arterial connection of the peritruncal coronary network by ingrowth of the coronary stems into the aortic wall at stage HH32 (ED7.5)[92,96]. Initial retroviral labeling experiments showing that the SMCs and adventitial fibroblasts of the coronary system originate from the PEO[63,97] were confirmed by several studies in quail-chicken chimeras[42,45,62,98]. Recruitment of SMCs and pericytes to the developing vessel network is primarily regulated by PDGF-B and its receptor PDGFRβ[99], expressed by coronary endothelium and attracted EPDCs, respectively[61]. TGFβ and serum response factor (SRF) are involved in SMC differentiation of the recruited EPDCs[57,59,60]. The role of EPDCs in coronary development was further studied by manipulation of the epicardial contribution to the developing heart, which resulted in hampered development of the coronary arteries. After complete PEO ablation, genetically directed lack or mechanical inhibition of epicardial outgrowth, the coronary vasculature fails to form[37,38,43,69,70,74,100], but also less severe epicardial manipulations will give rise to a lack of endothelial vessel stabilization with consequent maldevelopment of the coronary vasculature and absent or abnormal coronary ostia[54,69,73,101]. Formation of the coronary ostia is strongly associated with apoptosis in the peritruncal ring[102,103], which was shown to relate to the expression of FasL by EPDCs[69].

As to the origin of the endothelial component of the coronary arteries, seemingly conflicting results feed the debate. Despite reports on a common epicardial origin of the coronary endothelium and the coronary media and adventitia[78,104,105], we favor the view that only the SMCs and the fibroblasts of the coronary system are true EPDCs. The endothelium of the coronary vasculature is actually liver derived, since PEO transplants without a tiny piece of liver will never give rise to endothelial cell formation in the recipient heart[23]. The separate origins of the coronary endothelial and SMCs were also recognized in retroviral tracing experiments, which never showed labeling of both endothelium and SMCs in the same coronary segment[63]. Recently, reporter gene expression under control of the epicardium-specific GATA-5 reporter in the coronary smooth muscle layer, but not in the endothelium, provided additional evidence for a nonepicardial origin of the endothelial coronary component. The fact that endothelial (precursor) cells reach the heart via the PEO[22,23] and can be found there already at stage HH16/17 (ED2.5)[24,25] may well explain findings of a seemingly common epicardial origin of endothelium and mesenchymal coronary contributors in studies with proepicardial explants of similar developmental stages.

A recent study on quail-chicken chimeras revealed that, indeed, the proepicardium is a vehicle for the hemangioblasts, but that the cardiac lymph vessels that develop in close physical association with the coronary system are derived from a nonepicardial source of lymphangioblasts[106]. Probably the very same hemangioblast population gives rise to the erythrocytes in the subepicardial blood-forming islands that have been observed in avian embryonic hearts under normal[107] and pathological conditions[108].

### Valve Development

Embryonic valve development starts with the formation of the endocardial cushions in the still-primitive heart tube at the atrioventricular boundary and in the distal outflow tract. Stimulated by inductive signals such as BMP-2 from the myocardium, the endocardium of these regions undergoes EMT to deposit mesenchymal cushion cells in the underlying extracardiac jelly[109,110]. Further mesenchymal cells are added to the atrioventricular cushion tissue when EPDCs start to migrate in, which, in the avian embryos, is during the second influx of EPDCs from HH31 (ED7) onward[42,62]. The relative physical contribution of EPDCs to the atrioventricular valves seems to decrease in time, as at stage HH45 (ED19), only modest numbers of quail-derived EPDCs were found in quail-chicken chimeras[111]. This may indicate that EPDCs are necessary and present as local inductive cells only within a specific time window, or that the relative contribution of endocardially derived mesenchyme still increases after stage HH35 (ED9). This would not be in line with a hypothetical inhibitory action of EPDCs on endocardial EMT that was postulated earlier[62]. The expression of TGFβ1-3 in the proepicardium, epicardium, and isolated cells within the developing atrioventricular valves[56] might rather suggest that EPDC-derived TGFβ signaling would enhance EMT, and shift the delicate balance between endocardium and mesenchyme that is dominated by VEGF and TGFβ2[112] to an increase in mesenchymal cushion cells. Alternatively, the proportion of atrioventricular cushion cells derived from the epicardium may diminish during development by the influx of hematopoietic stem cells entering the cushion from the circulation[113,114].

From the colocalization of EPDCs with procollagen I in the developing atrioventricular cushions and fibrous heart skeleton, a morphogenetic role in the fibrous differentiation during valve development was postulated for EPDCs[62]. This is also indicated by aberrant cushion formation in embryos with defective epicardial development, which are either underdeveloped and too small[43,69,72,115], or immature and too big[52].

During valve formation, myocardial tissue initially replaces or grows into the cushion mesenchyme by a process called myocardialization[116,117]. In a later stage, this myocardium is replaced by or transdifferentiates into the fibrous tissue that eventually forms the fibrous valve leaflet. Subsequent delamination of the ventricular aspect of the atrioventricular cushion forms the loose valve leaflets of both the tricuspid and mitral valve in mammals[118,119,120], whereas in avian species, only the left atrioventricular valve delaminates[120].

Interestingly, there is a spatiotemporal correlation between the presence of EPDCs, the expression of the extracellular matrix molecule periostin, and the fibrous differentiation process of the atrioventricular valves (*cf*. [62] and [121,122,123]). We therefore hypothesize that EPDCs contribute to fibrous valve differentiation through the local production of periostin. *In situ* hybridization analysis showed that the epicardium, the EPDCs in the subepicardium, as well as dispersed cells in the myocardium synthesize periostin[122]. In recent literature, periostin emerges as a multipotent molecule involved in cardiomyocyte-fibroblast interactions[124] with an ambiguous role under pathological conditions. Whereas periostin has been reported to induce re-entry of cardiomyocytes into the cell cycle and to promote cardiac repair in the infarcted myocardium[125], other studies point at a profibrotic role for periostin after cardiac or vascular injury (e.g., [126,127]). With regard to valve development, periostin is likely to be instrumental through its ability to enhance fibrous differentiation of the extracellular matrix by its direct interaction with collagen I[128] and by mesenchymal condensation via increased mesenchymal cell invasion and matrix compaction[129]. Whereas an insufficient physical contribution of EPDCs to the atrioventricular cushion may explain the hypoplastic atrioventricular valves observed after nearly complete epicardial ablation[43,69,72,115], suboptimal levels of EPDC-derived periostin may explain the presence of immature and hyperplastic atrioventricular valves with loose mesenchyme that are observed after incomplete inhibition of epicardial outgrowth with a piece of eggshell membrane[52]. Analysis of epicardially inhibited embryos in our lab confirms a consequent decrease in periostin levels and thick abnormal atrioventricular cushions at HH35 (ED9; unpublished observations).

In proepicardial quail-chicken chimeras, EPDCs were hardly ever found in the developing semilunar valves[42], even when EPDCs had migrated massively to the peritruncal area (unpublished observation). Hence, other signaling cells than EPDCs are involved in the formation of the peritruncal part of the fibrous heart skeleton, including the aortic and pulmonary valves. Most likely, neural crest cells (see Fig. 1) fulfill this function in the semilunar valves[130] through, for example, the expression of Pinch1[131].

### Purkinje Fiber Differentiation

Some years before the important role of EPDCs in cardiac development became apparent, retroviral tracing studies revealed a cardiomyocyte origin of the peripheral ventricular conduction system, the Purkinje fibers[132]. In the chicken embryo, a part of the Purkinje fibers develop in association with coronary arterialization[133,134]. This is largely dependent on the hemodynamically regulated endothelial expression of endothelin-1 (ET-1) after cleavage from its pre-pro form by endothelin-converting enzyme 1[135,136,137]. The periarterial Purkinje fibers develop not only in close association with the coronary arteries, but also in close association with the colocalizing EPDCs[62]. The subendocardial Purkinje fibers develop at sites where EPDCs are present as well. Direct proof for the hypothesis that EPDCs contribute to the formation of the Purkinje fiber network[138] came from experiments in which proepicardial outgrowth inhibition caused hypoplasia of the Purkinje fibers in quail embryos[71]. EPDCs might be instrumental in Purkinje fiber development either by “priming” of cardiomyocytes to respond to the endothelial or endocardial ET-1 signal, or via direct ET-1 production by the EPDCs themselves[139].

Although the paradigm of a cardiomyogenic origin of the Purkinje fibers is still holding strong, it should be noted that the retroviral transductions of the surface myocardium in the retroviral tracing study[132] were performed at a developmental time point (HH19–21; ED3) at which the surface of the ventricle is already partly covered by epicardium[21], and tracings with injections before this time point did not give rise to labeling cells of the Purkinje fiber network[140]. The recent finding that mesenchymal stem cells, which are comparable to EPDCs to some extent[141], but do not differentiate into cardiomyocytes *in situ*[142,143,144], can differentiate into Purkinje fibers[145,146] further indicates that the common view in which the cardiomyocyte is the sole precursor for cells of the Purkinje fiber network may need refinement.

### Establishment and Maintenance of Myocardial Architecture

EPDCs are essential for normal myocardial architecture, which is evident from the numerous studies in which mechanical[43,69,70]or genetic manipulation[37,38,54,73,74,75,76,77] of epicardium resulted in a thin ventricular myocardium. There is a close spatiotemporal relationship between the influx of EPDCs into the myocardium and the onset of the formation of the ventricular compact zone[62]. Cardiomyocytes have been reported to develop in PEO explant cultures after stimulation with BMP-2[16]. However, these PEO explants consist of a mixed cell population, containing endothelial (precursor) cells, myocyte precursors from the sinus venosus region, and epithelial and mesenchymal epicardial cells. The latter are the source for EPDCs, which have never been shown to be capable of differentiation into cardiomyocytes *in vivo*[42,62]. For that reason, their influence on myocardial development must be a regulatory one via paracrine signaling routes and the secretion of extracellular matrix(–modifying) molecules. Equally important, they contribute to myocardial architecture by their physical presence as interstitial fibroblasts. Fibroblasts have been shown to constitute up to 70% of the myocardial wall[147,148], with species differences between rat and mouse related to size of the cardiac diameter and consequent wall tension[149]. Although a blood-borne origin of cardiac fibroblasts has been described[114,150], the interstitial myocardial EPDC distribution in quail-chicken chimeras indicates that a large proportion of the cardiac fibroblasts originates from the epicardium[25,62].

*In vivo* research on the role of interstitial EPDCs in myocardial development has been greatly frustrated by the lack of an adequate intrinsic marker for this specific set of differentiated fibroblasts. WT-1 and RALDH2 are both expressed in epicardium, subepicardial EPDCs just after EMT, and after initial invasion of EPDCs into the myocardium[51,52,53,151], but expression of these markers is lost in fully differentiated EPDCs. Only in quail-chicken chimeras can differentiated EPDCs be identified unambiguously by the quail-specific nuclear marker QCPN. A general use of the mouse mutants in which the epicardium-specific GATA-5 promoter drives the expression of LacZ[73] will help to unravel the role of interstitial EPDC in the formation of the ventricular compact zone.

Up to now, the signaling pathway of the vitamin A metabolite retinoic acid is the best described paracrine effector route by which EPDCs influence myocardial development. Initiated by the finding that mice deficient for the retinoic acid receptor RXRα showed a thin ventricular myocardium[75,76,89,152] in which the compact myocardium failed to form, subsequent *in vitro* studies showed that retinoic acid treatment releases a trophic factor from EPDCs that promotes cardiomyocyte proliferation via activation of intracellular PI3K and Erk signal transduction pathways[153,154]. The finding that the key enzyme in retinoic acid synthesis, RALDH2, is expressed in the epicardium and by EPDCs[52,151], and that epicardium-specific[73], but not myocardium-specific[155], RXRα knockout mice have a thin ventricular myocardium indicate that this instructive feature of EPDCs is regulated by an autocrine loop. FGFs are among the trophic factors that are up-regulated in the epicardium by retinoic acid[153,155,156]. In turn, FGF-2, -9, and -16, signaling to the myocardium through the FGF receptors 1 and 2, were shown to enhance cardiomyocyte proliferation[157,158,159]. Additional candidates that have been identified as factors involved in the cross-talk between myocardium and epicardium that are influencing myocardial architecture are ET-1[139,160], FOG-2[74], and erythropoietin[156].

Although extracellular matrix biology is essential for myocardial architecture during development and remodeling[161], only few studies have focused on the extracellular matrix as a signaling vehicle. In the RXRα knockout mouse, defective epicardial development was associated with an increase in fibronectin deposition[152]. The finding that the extracellular matrix protein periostin is expressed by fibroblasts and by mesenchymal cells that differentiate into fibroblasts[121,122] and colocalize with the presence of EPDCs — in the epicardium, the subepicardium, the atrioventricular valves, and in interstitial cells within the ventricle[62] — opens up a new avenue for the research on the role of EPDCs in myocardial development. The “periostin hypothesis” proposes that periostin promotes and sustains differentiation of the fibroblast phenotype, possibly also in relation to cardiomyocytes and EPDCs that are involved in the formation of the peripheral fibrous heart skeleton[114].

### EPICARDIUM-RELATED CONGENITAL HEART MALFORMATIONS

In the preceding sections, we have mentioned the association of EPDCs with valve development and maturation. Interestingly, a part of the EPDCs that migrate to the atrioventricular cushions are deposited in the subvalvular ventricular myocardium[62], at the site where it delaminates and eventually develops into the tendinous chords[118,119]. When impaired EPDC migration would cause incomplete delamination of the valve leaflet, this might present clinically as a tricuspid valve displacement towards the apex as seen in Ebstein's anomaly (recently reviewed[162]).

Similarly, EPDCs may very well be involved in the incompleted development of a fibrous insulation in the atrioventricular junction. We found that periostin expression in the subepicardium of the atrioventricular sulcus is likely to be involved in the shutting off of the atrioventricular connections between atrial and ventricular myocardium[163]. Persistence of these myocardial atrioventricular bridges may lead to pre-excitation of the ventricle, which is a characteristic of Wolff-Parkinson-White (WPW) syndrome[164]. Similar electrical bypasses consisting of functional myocardium were also found in a model for Mahaim tachycardia[165].

In addition, the punctate expression pattern of periostin mRNA in the ventricular myocardium[122] suggests that interstitial myocardial EPDCs also secrete periostin, and thus contribute to the integrity and architecture of the working myocardium. In this respect, it is interesting that the clinical manifestation of WPW syndrome is often coincident with Ebstein's anomaly[166] and that it can be accompanied by the myocardial abnormality referred to as ventricular noncompaction[162,167], in which the compact layer of the ventricles is maldeveloped. Defects reminiscent of this triangle of congenital heart malformations were also observed in the epicardium-deficient, FOG-2 knockout mouse. This develops tricuspid atresia and a thin myocardium, in addition to a double-outlet right ventricle and ventricular septal defect VSD

that might also be associated with defects in epicardial outgrowth. Also in embryos with genetically or epicardially inhibited PEO outgrowth from our lab, valve abnormalities, thin myocardium, double-outlet right ventricle, VSD[43,54,69], and persistent atrioventricular myocardial bridges (unpublished results) were apparent. Therefore, we propose that an epicardial outgrowth and/or differentiation deficit may well prove to be the common cause underlying the associated development of these congenital heart defects in man.

### ACKNOWLEDGMENTS

Ron Slagter and Jan Lens are kindly acknowledged for preparation of the figures. This work was funded partly by the Netherlands Heart Foundation (grant number NHS 2001B015 to H.L.-V.)

## MATERIALS AND METHODS SUPPLEMENT

### Antisense Ets Lentiviral Constructs

Three antisense Ets constructs were made using the control lentiviral sequence in which GFP expression was driven by the CMV promoter[1,2]. The CMV-GFP-asEts1/2 (asEts-1/2) construct harbors the same antisense Ets sequence as the retroviral CXasEtsIZ vector used in earlier studies[3,4]. The CMV-GFP-asEts-1 (asEts-1) construct contains a 562-bp-long antisense chicken Ets-1 sequence (cloned as a RT-PCR fragment from total chicken RNA; nucleotide +1834 to +2396[5]), which is highly specific for Ets-1 mRNA, whereas the CMV-GFP-asEts-2 (asEts-2) contains a 580-bp-long antisense chicken Ets-2 sequence (also cloned as an RT-PCR fragment from total embryonic cardiac chicken RNA, from nt +761 to +1341[6]), specifically targeting Ets-2 mRNA. The antisense cDNAs were cloned immediately downstream of the GFP-encoding sequence.

### Proepicardial Explant Culture and Transduction

PEOs were isolated from stage HH15–17 (ED2.5) quail embryos and allowed to adhere in Primaria culture wells (Falcon). Lentiviral preparations were added to the proepicardial explants to a final concentration of 500 ng p24 core protein/ml. After overnight incubation, the viral suspension was replaced with medium (M199-Hepes, supplemented with 10% inactivated fetal calf serum, insulin-transferrin-selenium-G [Invitrogen], penicillin, streptomycin, and gentamycin) and the explants were cultured for 7 days.

### Proepicardial Quail-Chicken Chimeras

Preparation of proepicardial quail-chicken chimeras and tissue processing was done as described before[7]. Attachment of the PEO and outgrowth of quail EPDCs in the chicken host was analyzed by staining with the quail-specific nuclear antigen QCPN. Only hearts in which more than 500 grafted quail EPDCs could be counted were used for analysis.

### Antibody Staining and Detection

Quail endothelial cells were identified by immunohistochemical or immunofluorescent staining with the QH-1 antibody (Hybridoma Bank) as described earlier[7]. SMCs were identified by staining with 1A4 antibody specific for α-smooth muscle actin (Sigma). Anti-GFP (Clontech, BD Biosciences, antibody JL-8) and quail-specific QCPN (Hybridoma Bank) were used to detect which proportion of the quail-derived EPDCs expressed the lentivirally encoded GFP marker. Conventional light microscopy after immunohistochemistry with anti-GFP and quail-specific QCPN and confocal microscopy after immunofluorescent staining with QH-1 were used to visualize the grafted EPDCs in stage HH35 (ED9) chimeras.
